# Atypical de Winter Presentation of Critical Left Anterior Descending Coronary Artery Occlusion

**DOI:** 10.7759/cureus.24724

**Published:** 2022-05-04

**Authors:** Aleesha Kainat, Noor Ul Ain, Hetal Boricha, Mahdin Gulzar, Eric J Dueweke

**Affiliations:** 1 Internal Medicine, University of Pittsburgh Medical Center McKeesport, Pittsburgh, USA; 2 Internal Medicine, Allama Iqbal Medical College, Lahore, PAK; 3 Cardiology, Heart and Vascular Institute, University of Pittsburgh Medical Center Presbyterian, Pittsburgh, USA

**Keywords:** de winter's syndrome, stemi, acute coronary syndrome (acs), interventional cardiology, primary percutaneous coronary intervention (pci), electrocardiogram (ecg/ekg)

## Abstract

A 69-year-old male presented with substernal chest pain that started a few hours earlier. On arrival, the patient was hemodynamically stable, and the physical examination was unrevealing. Laboratory workup revealed an elevated high-sensitivity troponin, and an initial electrocardiogram (ECG) revealed tall, symmetric T-waves with preceding minor concave ST-segment elevations less than 1 mm in the precordial leads (V1-V6) and 0.5 mm ST elevation in the aVR. Due to concerning ECG changes, the patient was treated for a possible non-ST-segment elevation myocardial infarction. A loading dose of aspirin and clopidogrel was given and a heparin drip was initiated. However, the patient’s chest pain persisted requiring multiple sublingual nitroglycerin tablets. Later, on further review of the ECGs, the presence of de Winter T-waves was noted and led to activation of the catheterization laboratory, and an urgent left heart catheterization (LHC) was done. LHC revealed a critical 90% occlusion of the left anterior descending artery, and a drug-eluting stent was placed. The patient had a good recovery thereafter. This case emphasizes the rarity of the case and lack of awareness about the atypical de Winter pattern that is considered to be an ST-segment elevation myocardial infarction equivalent. Failure to recognize this can potentially lead to delayed intervention.

## Introduction

Electrocardiogram (ECG) remains one of the most important diagnostic tests for various life-threatening conditions due to its accuracy (specificity of 88-96%, and a positive predictive value of 88%) [1], accessibility, and convenience. The mainstay of treatment for acute coronary syndromes is dependent on timely ECG interpretation of high-risk characteristics such as ST elevations or a new left bundle branch block in an appropriate clinical context. With the widespread utility of ECG, most physicians are trained to interpret high-risk features and offer immediate medical assistance. A significant challenge occurs when uncommon ECG patterns are encountered which represent underlying high-risk conditions, often leading to misinterpretation, delay in providing timely care, and an increase in mortality. De Winter syndrome is one such phenomenon that was first described by de Winter et al. as a diagnostic for occlusion of the left anterior descending (LAD) branch of the left main coronary artery [2]. The authors described the ECG pattern where the J-point of the precordial leads was lowered by 1-3 mm, and the ST segment appeared as an upsloping depression, followed by positively symmetrical T-waves, associated with a slight elevation of aVR. They found that this ECG pattern is consistent with the proximal LAD conclusion in 2% of patients who presented with acute coronary syndromes. However, more recent studies have described atypical variants of the de Winter pattern that did not strictly follow the classical de Winter criteria, and are still considered and treated as ST-segment elevation myocardial infarction (STEMI) equivalents [3]. In our case, the typical presence of tall symmetrically positive T-waves in precordial leads was associated with concave ST-segment elevations rather than depressions and a subtle aVR elevation. This led to a misdiagnosis as a non-ST-elevation myocardial infarction leading to delayed percutaneous coronary intervention (PCI) which revealed a subtotal LAD occlusion.

## Case presentation

A 69-year-old Caucasian male presented to the hospital with crushing substernal, non-pleuritic, and non-positional chest pain, 7/10 in intensity, radiating to bilateral shoulders that had started two hours earlier while he was driving to work. He also endorsed marked diaphoresis and generalized weakness associated with chest pain. He had never experienced a similar episode of chest pain before this incident. His medical history was significant for long-standing inadequately controlled hypertension, hyperlipidemia, and coronary artery disease, which was evidenced by a percutaneous coronary angiogram done a few years ago that revealed diffuse mild-to-moderate disease in coronaries (30-50% stenosis) not requiring PCI. His family history was significant for myocardial infarction in both parents at the age of 60. Upon presentation, he was noted to be afebrile, hypertensive with a blood pressure of 185/88 mmHg, heart rate of 76 beats per minute, and saturating 98% on room air. His physical examination revealed a well-appearing male in acute distress due to chest pain and lungs clear to auscultation bilaterally. A cardiovascular examination revealed normal heart rate and rhythm without any murmur, rub, or gallops. His abdomen was soft and non-tender, without pedal edema. Finally, a neurological examination was unremarkable.

Investigations

**Figure 1:**
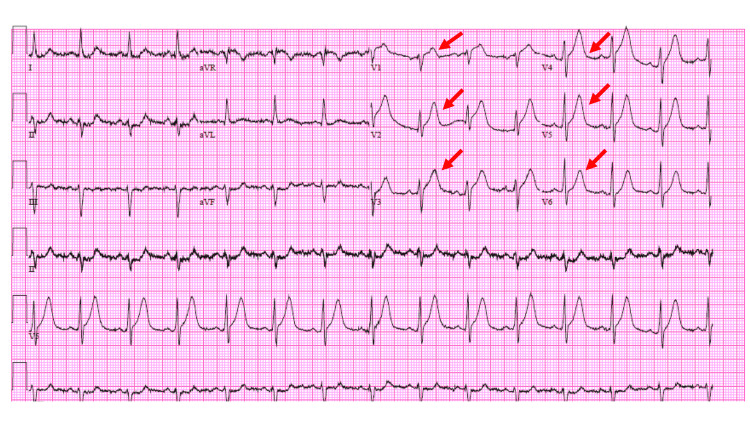
Electrocardiogram on presentation revealing de Winter T-waves.

Initial laboratory workup showed a high-sensitivity troponin elevated at 387 ng/L (normal values: less than 59 ng/L), which later trended up to 14,000 ng/L, lactic acidosis of 4.1 mMol/L (0.5-1.9 mMol/L), hyperlipidemia with a total cholesterol of 206 mg/dL (normal range: less than 200 mg/dL), low-density lipoprotein elevated at 133 mg/dL (desired range: less 70 mg/dL), and triglycerides elevated to 173 mg/dL (normal range: less than 150 mg/dL). An ECG obtained on presentation was concerning for tall, prominent, symmetrical T-waves in the precordial leads with associated minor concave ST-segment elevations, and less than 0.5 mm ST elevation in aVR (Figure 1). A computed tomography scan of the chest did not reveal any acute pulmonary embolism; however, it did reveal mild aneurysmal dilatation of ascending thoracic aorta to 4.2 cm which was stable when compared with imaging obtained 10 years ago.

Treatment

**Figure 2:**
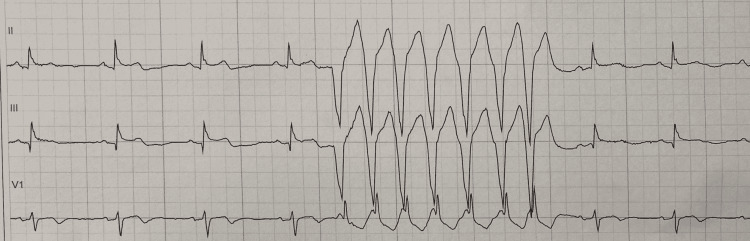
Non-sustained ventricular tachycardia noted on telemetry.

**Figure 3:**
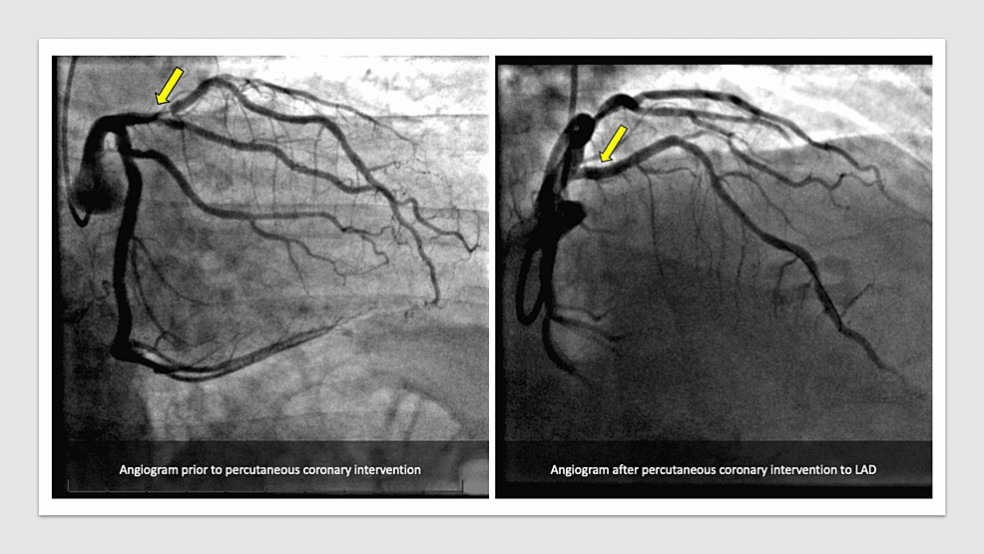
Images from left heart catheterization before and after percutaneous coronary intervention.

The emergency department physician and the on-call cardiologist reviewed the ECG, and a working diagnosis of the acute coronary syndrome was postulated with non-ST-segment elevation myocardial infarction (NSTEMI) as the most likely possibility. The patient was administered sublingual nitroglycerin with some relief of his chest pain. He was also treated with a loading dose of aspirin 325 mg, clopidogrel 300 mg, and was started on a heparin drip. The patient continued to have chest pain episodes through the night requiring multiple sublingual nitroglycerin tablets, and subsequently a nitroglycerin paste administration. The next morning, a review of the patient’s overnight telemetry revealed episodes of non-sustained ventricular tachycardia (Figure 2). The high-sensitivity troponin trended up to 14,000 ng/L. The cardiology team evaluated the patient the next morning and activated a STEMI alert based on their interpretation of the ECG concerning for an atypical de Winter pattern of STEMI. The patient was taken to the catheterization laboratory where he underwent left heart catheterization (LHC) which revealed a subtotal occlusion of LAD, with successful drug-eluting stent deployment (Figure 3) and optimal apposition of the stent which was confirmed by intravenous ultrasound during the procedure. His left circumflex disease revealed a 40% disease in the proximal part of the vessel. His right coronary artery was without any significant disease. The patient was recommended dual-antiplatelet therapy with aspirin and ticagrelor for one year and was started on high-intensity statin therapy, along with a beta-blocker. A subsequent transthoracic echocardiogram revealed a non-dilated left ventricle with hypokinetic apical inferior wall and apical septum with a low normal left ventricle ejection fraction of 50-55%.

Outcome and follow-up

The patient was evaluated at a follow-up telemedicine appointment one-month post-discharge. He did not report any anginal episodes, endorsed compliance with his medications, and did not report any side effects. He was also actively participating in cardiac rehab and had returned to his baseline level of activity.

## Discussion

Early and prompt PCI with reperfusion remains the gold standard of treatment for STEMI and high-risk non-STEMI patients [4]. Many ECG patterns have been recognized as STEMI equivalent such as a new left bundle branch block [5] and are considered cardiac emergencies in the appropriate clinical context. De Winter syndrome is one such criterion that was described in 2008 by de Winter et al. as a STEMI equivalent in nearly 2% of the patients presenting with LAD occlusion [2]. They studied 1,536 patients who presented for an acute coronary syndrome and subsequent evidence of LAD occlusion, 30 of whom did not have typical ST elevation but presented with 1-3 mm upsloping ST-segment depression at the J-point in leads V1 to V6, followed by positive symmetrical T-waves and a 1 to 2-mm ST elevation in lead aVR. Since then, this pattern has been reported in multiple case reports [6-8], where patients had these typical ECG characteristics and were found to have significant LAD occlusion. This pattern has positive predictive values of 95% to 100%, as described in the literature, based on three respective diagnostic studies [9]. Despite the available literature, it remains an uncommon finding and can be easily overlooked when patients present to the emergency room and causes a delay in activation of the catheterization laboratory. Although initially de Winter pattern was considered a static condition that did not evolve into ST-segment elevation [2,10], it was later described that de Winter ECG could evolve into ST-segment elevations [8,11]. It has also been described that ST elevations can evolve into de Winter ECG pattern [12]. Hence, based on the existing data, two patterns of de Winter have been proposed [3,13], namely, static presentation with persistent J-point depression before revascularization, and a dynamic pattern that may evolve into ST elevation. Furthermore, recent reports of atypical presentations of de Winter have been published. Paixão-Ferreira et al. reported an atypical presentation of de Winter with a downsloping ST-segment depression in leads V2-V3 (and, to some extent, in V1) with an absence of significant ST-segment elevation in lead aVR [14]. Similarly, another case report described a de Winter pattern characterized by prominent T-waves and downsloping ST-segment depression in leads V3-V6, along with mild ST-segment depression in the inferior leads in a young male [13]. Interestingly, an acute right coronary artery occlusion was described with a typical de Winter pattern in the inferolateral leads with upsloping ST-segment depression at the J-point continuing into positive symmetrical T-waves in leads II, III, aVF, and V4-V6, coupled with ST-segment elevation in lead aVR [15]. Yalta et al. described these atypical presentations and raised the question of whether atypical de Winter might be more prevalent and have a higher propensity to be missed as they do not conform to the classical pattern [3].

In our case, the patient did not have the preceding upsloping ST-segment depressions with positive, tall, symmetric T-waves rather concave elevations which were deemed non-specific changes, and the patient was managed as an NSTEMI. In light of the existing data, we postulate that in our case by the time our patient presented to the emergency room, the de Winter ST depressions had already been evolving to the concave ST elevations, leading to an atypical presentation of the de Winter pattern rather a classical presentation. We also hypothesize that the tall, symmetric, T-waves seem to be the most characteristic of de Winter syndrome as well as ischemia as that seems to be the persistent finding in cases that report atypical presentations [3,13,15]. We contribute to the existing data by reporting a case of critical LAD stenosis which presented as an atypical de Winter pattern and led to a delayed diagnosis and attest to the lack of awareness of this ECG pattern among physicians. Fortunately, in our case, it was rectified soon, and the patient received an intervention with a good outcome.

Although the underlying electrophysiological explanation for these ECG changes is not entirely known, few theories have been proposed. These findings have been attributed to certain anatomical variants such as an anatomical variant of Purkinje fibers with endocardial conduction delay [2], preexisting ECG changes [3], or the existing collateral blood supply which prevents the myocardium from transmural ischemia, hence preventing ST-segment elevation [10]. Alternatively, lack of activation of sarcolemmal ATP-sensitive potassium (KATP) channels by ischemic depletion of ATP can also explain the absence of ST elevation, evidenced by the acute ischemia models of KATP knockout animals [16].

## Conclusions

De Winter syndrome is an uncommon ECG pattern considered to be a STEMI equivalent whose misdiagnosis can lead to delayed PCI and increasing mortality. In a proper clinical setting obtaining serial ECGs and early cardiology referral where challenging ECGs are encountered can lead to favorable outcomes; however, atypical de Winter presentation poses a challenge. De Winter T-waves and high-risk patient factors such as refractory chest pain and episodes of non-sustained ventricular tachycardia when encountered should prompt an early invasive strategy.

## References

[1] Leisy PJ, Coeytaux RR, Wagner GS (2013). ECG-based signal analysis technologies for evaluating patients with acute coronary syndrome: a systematic review. J Electrocardiol.

[2] de Winter RJ, Verouden NJ, Wellens HJ, Wilde AA (2008). A new ECG sign of proximal LAD occlusion. N Engl J Med.

[3] Yalta K, Yetkın E, Taylan G (2021). de Winter pattern: is it always so typical?. Rev Port Cardiol (Engl Ed).

[4] Rokos IC, French WJ, Mattu A, Nichol G, Farkouh ME, Reiffel J, Stone GW (2010). Appropriate cardiac cath lab activation: optimizing electrocardiogram interpretation and clinical decision-making for acute ST-elevation myocardial infarction. Am Heart J.

[5] Asatryan B, Vaisnora L, Manavifar N (2019). Electrocardiographic diagnosis of life-threatening STEMI equivalents: when every minute counts. JACC Case Rep.

[6] Lu B, Fu D, Zhou X, Gui M, Yao L, Li J (2020). A middle-aged male patient with de Winter syndrome: a case report. BMC Cardiovasc Disord.

[7] Yuanyuan X, Zhongguo F, Bao XU, Shenghu HE (2020). [de Winter syndrome, an easily ignored but life-threatening disease: a case report]. Nan Fang Yi Ke Da Xue Xue Bao.

[8] Verouden NJ, Koch KT, Peters RJ (2009). Persistent precordial "hyperacute" T-waves signify proximal left anterior descending artery occlusion. Heart.

[9] Morris NP, Body R (2017). The de Winter ECG pattern: morphology and accuracy for diagnosing acute coronary occlusion: systematic review. Eur J Emerg Med.

[10] de Winter RW, Adams R, Verouden NJ, de Winter RJ (2016). Precordial junctional ST-segment depression with tall symmetric T-waves signifying proximal LAD occlusion, case reports of STEMI equivalence. J Electrocardiol.

[11] Fiol Sala M, Bayés de Luna A, Carrillo López A, García-Niebla J (2015). The "de Winter pattern" can progress to ST-segment elevation acute coronary syndrome. Rev Esp Cardiol (Engl Ed).

[12] Zhao YT, Wang L, Yi Z (2016). Evolvement to the de Winter electrocardiographic pattern. Am J Emerg Med.

[13] Velibey Y, Genç D, Inan D, Tezen O (2019). The de winter electrocardiographic pattern: what else do we need to learn?. Int J Cardiovasc Acad.

[14] Paixão-Ferreira M, Rocha AR, Piçarra B, Pais J, Carvalho J, Aguiar J (2020). De Winter pattern: an ST-elevation myocardial infarction equivalent. Rev Port Cardiol (Engl Ed).

[15] Tsutsumi K, Tsukahara K (2018). Is the diagnosis ST-segment elevation or non-ST-segment elevation myocardial infarction?. Circulation.

[16] Li RA, Leppo M, Miki T, Seino S, Marbán E (2000). Molecular basis of electrocardiographic ST-segment elevation. Circ Res.

